# Targeted Biofeedback Training to Improve Gait Parameters in Subacute Stroke Patients: A Single-Blind Randomized Controlled Trial

**DOI:** 10.3390/s24227212

**Published:** 2024-11-11

**Authors:** Dmitry V. Skvortsov, Sergey N. Kaurkin, Galina E. Ivanova

**Affiliations:** 1 Center for Brain and Neurotechnology, Moscow 117513, Russia; 2 Research and Clinical Centre, Moscow 107031, Russia

**Keywords:** cerebral stroke, hemiparesis, gait, rehabilitation, biofeedback

## Abstract

Biofeedback (BFB) is a rehabilitation method, which, among other things, is used for the restitution of motor and gait function. As of now, it has become technically feasible to use BFB training based on target gait parameters to improve the gait function in stroke patients. The walking patterns of stroke patients are generally characterized by significant gait phase asymmetries, mostly of the stance phase and the single stance phase. The aim of the study was to investigate the restoration of gait function using BFB training with gait phases as feedback targets. The study included two patient groups, each of 20 hemiparetic patients in the subacute stage of stroke and a control group of 20 healthy subjects. Each patient group received BFB training with either stance phase or single stance phase as the feedback target, respectively. The patients received a total of 8 to 11 training sessions. Assessments based on clinical scales and gait analysis data (spatiotemporal, kinematic, and EMG parameters) were performed before and after the training course. The score-based clinical assessments showed a significant improvement in both patient groups. According to the assessments of gait biomechanics, the subjects in the Single Stance Phase group had significantly more severe dysfunctions. In both patient groups, the unaffected limb responded to the BFB training, while the stance phase significantly changed after training in the unaffected limb only. The other patient group, trained using the single stance phase as the feedback target, showed no changes in the target parameter either in the affected or in the contralateral limb. The clinical and instrumental assessments showed different, non-equivalent sensitivity. The results of the study demonstrated the possibility to use targeted BFB training to improve walking function. However, a significant effect of such training was only observed with stance phase as the target parameter. A response to training was observed predominantly in the unaffected limb and facilitated the desired increase in the functional ability of the paretic limb. Training based on stance phase as the target parameter is probably preferable for the patient population under study.

## 1. Introduction

Biofeedback (BFB) technology is widely used in rehabilitation. BFB-based methods can be used to improve control of certain physiological functions. The patient receives the possibility to recognize some specific physical signs and change them. The advantage of the method is that it uses the body’s own resources to restore the function. The method is physiological and has few contraindications. It is non-invasive and combines well with other treatments.

The use of BFB technology to improve walking function has a relatively short history. A detailed review by Spencer et al. [1] rated its use as promising. The authors classified BFB parameters into traditional types accepted in gait biomechanics: spatiotemporal, kinematic, kinetic, and electromyographic (EMG). The basic criteria of BFB training of walking in patients with various disorders remain the subject of research. A considerable part of the relevant studies raises questions rather than offers comprehensive BFB protocols [1,2,3]. Although the biomechanical gait parameters are well known and markedly standardized, only a few of them can be measured by relatively simple means. These include the gait cycle time, cadence, stride length, and some others. Therefore, they were first used as target parameters in BFB training, and are still widely used as such now [4]. However, other parameters—such as the stance phase, single stance phase, swing phase, and particular joint amplitudes—cannot be easily measured. The classical video motion capture or force platforms are rather complex and expensive. BFB technology is based on the fact that the trained parameter must be recorded in real time and with sufficient accuracy and frequency [2,3,5,6]. At the same time, the operation speed of the entire system should be enough not only to capture the parameters, but also to process and display them to the patient. The recent development of wearable technologies has offered portable sensors that can be used not only for diagnosis, but also for rehabilitation [1,7].

Stroke is one of the diseases that significantly impair gait symmetry [8,9,10]. Stroke survivors are characterized by a significant asymmetry of two important parameters of the gait cycle (GC): the stance phase and its most affected part, the single stance phase [8]. On the paretic side, both parameters are significantly shorter than on the unaffected side. The single stance phase appears to be the most difficult parameter to train and the one that limits recovery the most, since during this phase only one (paretic) leg is on support. The entire bodyweight falls on the paretic limb. It is responsible for support, balance and propulsion.

BFB technology has a number of advantages for restoring walking function because it mobilizes the body’s own resources. The most attractive, in our opinion, is the possibility of training based on a single selected parameter, which has become technically feasible, e.g., using inertial sensors [1]. In the review by Spencer et al. [1], it is this technology that was identified as currently the most advanced, capable of significantly expanding the potential of BFB training of walking, including using telehealth and home-based methods. The use of inertial technology and artificial intelligence technology has made it possible to use biomechanical parameters of gait, such as stance phase and single stance phase, for biofeedback in a very low-cost and practically convenient way [11].

There were some earlier studies of using the stance phase as a BFB training parameter, but we found no such studies for the single stance phase.

One of the latest analytical reviews on the use of targeted BFB training based on biomechanical gait parameters in patients with cerebral stroke was conducted by Spencer et al. [1]. The authors noted the confusing results of prior studies with various target parameters for gait retraining in cerebral stroke survivors, yet such training definitely improved the gait function.

Studies by Beg et al. [12] and Nagano et al. [13] used a target parameter from the general category: foot clearance of the paretic limb. The subjects were provided with direct information, i.e., the foot trajectory. The real-time capture and visual presentation of the foot trajectory with video analysis systems is a rather challenging task. Khallaf et al. [14] used a feedback target from E-med pedography with a visual presentation of information to the subject. Kim et al. [15] conducted a similar study where the sensor-captured pressure under foot during walking was visualized and presented to the patients.

Spatiotemporal BFB training with stride length or width as the target parameter showed confusing outcomes in cerebral stroke patients [16,17,18]. Brasileiro et al. [19] reported no increase in stride length when using it as the target parameter. Stride width is used as the target parameter even less often [20]. In a high-quality randomized controlled study, Druzbicki et al. [21] found no significant improvement in gait quality using BFB training with stride length as the feedback target versus conventional treadmill training. An earlier study [18], however, demonstrated a positive effect of similar training. Another study [22] showed that training based on visual feedback is likely to have a favorable effect in patients in the subacute stage of ischemic cerebral stroke.

A study [9] evaluated a relatively short BFB training in cerebral stroke patients in the subacute stage. The total training course did not exceed 3 weeks and, on average, included 10 training sessions with stance phase as the target parameter. The results of the study did not allow an unambiguous conclusion, and further research is needed. Kantan et al. [23] also used inertial sensor technology, but with swing phase as the feedback. That was an exploratory study, and the effectiveness of the approach should be further evaluated in the future.

The objective of the present study was to investigate the feasibility of restoring the gait function in subacute post-stroke patients using BFB training with stance phase and single support phase as the feedback targets.

## 2. Materials and Methods

### 2.1. Participants

The study was conducted in 2020–2023 in the Biomechanics Laboratory at the Center for Brain and Neurotechnology, Moscow, Russia. The single-blind randomized controlled study enrolled patients at the second stage of medical rehabilitation at the center. The study included two patient groups (20 subjects each) after a first hemispheric ischemic stroke and a control group of 20 healthy subjects. The patients received courses of BFB training based on either stance phase or single support phase as target parameters in the first or second patient group, respectively.

The study was registered 8 March 2024 at Clinicaltrials.gov with number NCT06299943.

*Inclusion Criteria*: age < 75 years; primary ischemic stroke; cerebral hemispheric lesion; functional ability to walk for at least 5 min without using external means of support; no mental impairment; no sensorimotor aphasia; muscle tone in the limbs not exceeding grade 2 according to Modified Ashworth Scale; no history of orthopedic and neurological pathology; and absence of pronounced pain syndrome.

*Exclusion Criteria*: signs of orthostatic hypotension during training; patient’s desire to withdraw from the study; and worsening neurological deficit.

*Study Groups*: There were two patient groups and a healthy control group in the study, with 20 subjects each. All patients received a combined treatment which included a conventional rehabilitation course, the same for all patients, and BFB training, which differed between the patient groups. The patient group that received training with stance phase as feedback target (Stance Phase Group, SPG) included 15 males and 5 females; 9 of the patients had right, and 11 had left hemisphere brain damage. The other patient group was trained based on single support phase (Single Support Phase Group, SSPG) and included 10 males and 10 females, with 11 and 9 of them having right or left hemisphere brain damage, respectively. Details are given in Table 1.

There was no statistical difference between the SPG and SSPG groups in subjects’ age, height, weight, and days after stroke.

All patients received conventional therapy according to clinical guidelines. It was not possible to exclude this part of rehabilitation. Thus, the two groups differed only in the BFB target parameter. The null hypothesis was that a comparison of the target parameter before and after the BFB training course would reveal a difference in the trained parameter in each group.

Clinical Condition Assessment: The degree of independence in physical activities of daily living was assessed using the Barthel Index [24], the Rivermead Mobility Index [25], and the Functional Independence Measure (FIM) [26].

The severity of the patient’s condition was assessed using the Modified Rankin Scale (mRS) [27]. The Rehabilitation Routing Scale (RRS) was used to plan their medical rehabilitation [28].

Gait assessments were based on the Timed Up and Go Test (TUG) [29], the Hauser Ambulation Index [30], and the Berg Balance Scale to assess the patient’s balance [31].

The Medical Research Council Weakness Scale was used to assess the muscle strength in the lower limbs [32].

### 2.2. Instrumental Gait Analysis

An objective assessment of the gait function was performed instrumentally using a Steadys gait analysis system (Neurosoft, Ivanovo, Russia). Seven inertial sensors were attached with elastic retaining bands to the subject’s sacrum, outer side of each thigh, outer side of each lateral malleolus, and each foot instep. Each sensor captured amplitudes of the respective joints and their functional EMGs. Disposable surface electrodes (Mederen, Tel Aviv-Jaffa Israel) were applied.

Each of seven inertial sensors had a wireless connection with a computer via a Wi-Fi interface. The neural network for gait cycle detection was trained to mark initial contact time based on navigation data [11].

The biomechanical gait parameters were evaluated while the subject was walking a distance of 10 m at a comfortable speed. At the end of the distance, the subject made a 180-degree turn and continued moving. The neural network operated in real time and excluded all unsteady or significantly different steps from further analysis. The test finished when 40 gait cycles were recorded for each lower extremity.

In accordance with the existing standard analysis of gait biomechanics [10,33,34], we recorded spatial and temporal parameters, joint kinematics, as well as envelope EMG of the muscles. Force platforms were not available for this study.

During the study, the neural network of the software captured gait cycles (GCs) for each lower limb and calculated other GC parameters based on the captured data. Spatiotemporal biomechanical parameters were recorded for subsequent analysis. The temporal parameters included gait cycle (GC) duration, in seconds; cadence or stride rate, in steps/min; foot clearance (Cl), in cm; gait speed (V), in km/h; and stride length (SL), in cm. Individual time periods of GC (measured as % from GC): stance phase (SP), single support phase (SSP), and total period of double support phase (DSP).

Kinematic parameters were recorded for the lower limb joints—hip, knee, and ankle—in the sagittal plane (flexion–extension). The software automatically generated goniograms for each joint in a gait cycle format. Maximum amplitude over GC was recorded in the hip joint (HA, degrees). For the knee joint, the recorded variables included first flexion amplitude (Ka1), extension amplitude (Ka2), and swing flexion amplitude (Ka3). For the ankle joint, maximum amplitude (AA) over GC was analyzed.

Maximum bioelectric activity of muscles over GC, in μV, was recorded for the tibialis anterior (TA), gastrocnemius (GA), quadriceps femoris (QA), and the hamstring (HM).

### 2.3. BFB Training Technique

The Steadys system has the functionality for targeted BFB gait training based on selected parameter. During such training (walking in a virtual environment with a specified task), the target parameter for each lower limb was presented in the form of a bar indicating current and desired values. If the subject was unable to perform the task, the range of desired values expanded and the movement speed in the virtual environment slowed down. If the task was successfully completed, the desired range was narrowed and the difference in mean values between the two limbs decreased in accordance with the algorithm used. The automatic training algorithm was configured so that, in the case of successful completion, the range of change was adjusted towards a greater symmetry of the parameters. The patients were not informed which specific parameter was trained. The subject’s values beyond the desired range of target parameter were displayed in red, while those within the range were displayed in green. Moreover, the subjects did not know which specific parameter was displayed on the screen.

The BFB training sessions were identical in both groups, with the only difference in the target parameter.

There is still no consensus on the optimal number of training sessions, or their duration and intensity for the studied patient population [1]. In this study, a training session normally continued until the patient became tired. Signs of regression of trained parameters were also taken into account: lack of improvement in the trained parameter, decrease in the rhythm of movement, or increase in the range of the trained parameter (all these data were displayed on the operator’s monitor).

The number of training sessions was 8–11 (9.2 ± 2.1) in the group that received training based on stance phase (SPG) and 8–11 (9.6 ± 0.9) in that with single support phase (SSP) as target parameter, without any statistical difference between the two patient groups.

The mean training session duration was 17.5 min (ranging from 15.1 to 20.4) and 19.0 min (13.0 to 25.0) in the SPG and SSPG groups, respectively. There was a significant difference in mean training session duration between the groups, with a longer training time in the SSPG group (*p* < 0.05).

Gait speed was measured on a treadmill Runner RHC500 (RHC500 Treadmill, Air Machine S.r.l., Cesena, Italy) [9,35]. The gait speed during the training sessions varied based on the patient’s comfort and the training safety criteria. The mean gait speed was 1.1 ± 0.5 (ranging from 0.3 to 2.1) km/h and 1.0 ± 0.4 (0.3 to 1.7) km/h in the SPG and SSPG groups, respectively, without any statistical differences between the patient groups.

In this study, the training session continued automatically until the patient was tired. In addition, the training parameter’s regression was taken into account: a lack of change in the trained characteristics, the decrease in the rhythm of movement, and the increase in the limits of change in the trained characteristics (which were displayed on the operator’s monitor).

### 2.4. Statistical Analysis

The obtained data were processed with the Statistica 12 software package using standard ANOVA methods to calculate the means and the standard deviations. The significance of differences was assessed using the Wilcoxon–Mann–Whitney test with *p* < 0.05. Pairwise comparisons were performed across paretic and non-paretic limbs. The number of patients in each group was determined so as to allow a valid statistical analysis and taking into account external factors that could not be changed. The latter were, first of all, the patient selection criteria used at the study center and a relatively small number of eligible patients in one year.

## 3. Results

The results of the score-based assessments of patient functional outcomes and independence in daily life activities are presented in Table 2. The provided treatment resulted in significant improvements as assessed with all scales used in the study (*p* < 0.05).

Both groups showed no statistically significant differences as assessed using all clinical scales except for the Berg Balance Scale. The Berg Balance Scale scores in the SPG group were significantly higher than in the SSPG group (*p* < 0.05), both before and after treatment. The study treatment resulted in a significant improvement in both patient groups as assessed using all scales used (*p* < 0.05).

**Figure 1:**
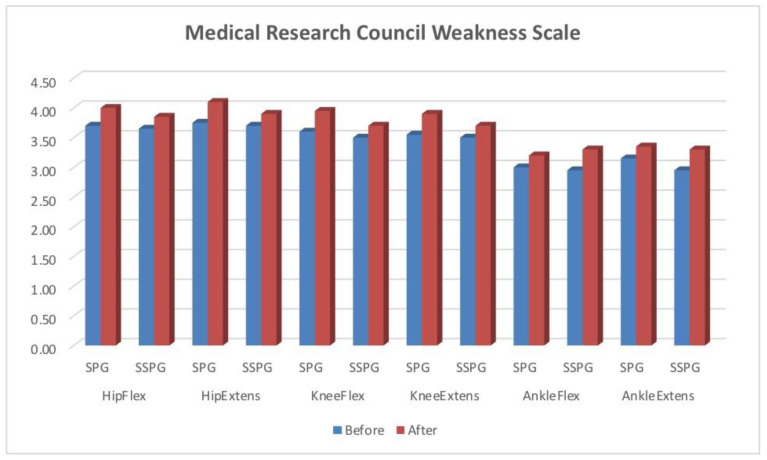
Muscle function assessments based on Medical Research Council grading system, before and after treatment. The vertical axis shows the MRC scale. Abbreviations: HipFlex—hip flexors, HipExtens—hip extensors, KneeFlex—knee flexors, KneeExtens—knee extensors, AnkleFlex—ankle flexors, AnkleExtens—ankle extensors, SPG—group trained with stance phase as target parameter, SSPG—group trained with single support phase as target parameter.

The results of dynamic assessments based on the Medical Research Council Weakness Scale are presented in the chart (Figure 1).

According to this scale, for all muscle groups, in both patient groups, the results were not statistically different from those before the treatment, i.e., the groups were also homogeneous when assessed using this scale. The delivered treatment resulted in a significant improvement of all parameters in both groups (*p* < 0.05).

The output of the spatiotemporal gait analysis is presented in Table 3.

In the two patient groups, all parameters, both before and after treatment, significantly differed from the control.

In the SSPG group, paretic limb clearance increased significantly after treatment.

In both patient groups, before and after treatment, the gait speed was significantly lower than in the control. In the SPG group, the V significantly increased after treatment.

In the SSPG group, the SL was significantly lower than in the SPG group, both before and after treatment. In both groups, the post-treatment SL was significantly higher than the initial one.

In the patient groups, both before and after treatment, the SP of the paretic and contralateral limbs was significantly higher than in healthy control.

In the SPG group, the pre-treatment SP of the contralateral limb as well as both pre- and post-treatment SP of the paretic limb were significantly higher than in the control. In both patient groups, the pre-treatment SP of the paretic limb was significantly lower than that of the contralateral limb. In both patient groups, the post-treatment SP of the contralateral limb decreased significantly compared to before the treatment. The post-treatment SP of the paretic limb significantly increased in the SPG group, while it significantly decreased in the SSPG group compared to pre-treatment values.

The pre- and post-treatment SSP of the paretic limb was significantly lower than those of the contralateral limb and of control. In the SSPG group, the pre-treatment SSP of the contralateral limb was significantly lower than that of the SPG group. In the SPG group, the post-treatment SSP of the paretic limb increased significantly compared to the pre-treatment value.

In both patient groups, the DS phase before and after treatment increased significantly on both sides compared to that in healthy controls. In the SSPG group, the pre-treatment DS phase on the paretic and contralateral sides was significantly increased compared to that in the SPG group.

The kinematic parameters for the hip, knee, and ankle joints are presented in Table 4.

In the SPG and SSPG groups, the amplitude of the contralateral limb hip joint was significantly lower than in healthy controls, both before and after treatment.

The pre- and post-treatment amplitude Ka1 in both lower limbs in both patient groups was significantly lower than in healthy control. In the SPG group, the Ka1 amplitude of the paretic limb after treatment was significantly higher than before it.

In the SSPG group, the post-treatment Ka2 of the paretic limb was significantly higher than that of the contralateral limb. In the same group, the Ka2 of the paretic limb before and after treatment was significantly higher than in the SPG group. In the SPG group, the pre-treatment Ka2 of the contralateral limb was significantly higher than that of the paretic limb. In the SPG group, the pre-treatment Ka2 of the contralateral limb was significantly higher than that of the paretic one and it increased significantly in the paretic limb after treatment.

In the SPG and SSPG groups, the swing amplitudes (Ka3) of both limbs before and after treatment were significantly lower than in healthy controls. In the SSPG group, the post-treatment Ka3 of the contralateral limb was significantly lower than in the SPG group. In the SSPG group, the Ka3 of the paretic limb after treatment was significantly higher than before treatment.

In the SPG and SSPG groups, the pre- and post-treatment amplitudes of the ankle joints in both lower limbs were significantly smaller than in healthy control. No statistically significant differences were found after treatment.

The results of the EMG activity study are presented in Table 5.

In both patient groups, the pre-treatment electrical activity of most muscles on the paretic side was significantly lower than in healthy controls and on the contralateral side. The only exception was for the quadriceps femoris, with its EMG activity similar to control. No significant changes in muscle activity were found after the treatment course.

## 4. Discussion

Two patient groups did not differ significantly in terms of the subjects’ clinical condition as assessed before the rehabilitation treatment. Thus, according to the clinical assessment tools, both groups were clinically comparable. Many studied parameters significantly changed as a result of the study treatment. The observed changes in the patient groups were comparable in their nature and magnitude. Assessments of patient condition by clinical scales showed similar results. Only the Berg Balance Scale scores reliably indicated that subjects in the SSPG group had more functionally severe conditions. The existence of a direct relationship between the functional indicators of balance and gait was noted in several instrumental studies [36,37]. Another study [38] demonstrated a high sensitivity of the Berg Balance Scale.

The instrumental gait assessments in our study showed that changes noted in both patient groups versus healthy controls were characteristic of a typical gait variant in hemiparetic individuals [9,39,40]. There were no significant differences between the patient groups in terms of the GC and CL. However, the Cl, V, GC, and SL in the SSPG group were significantly lower than in the SPG group. These were clear indicators that patients in the SSPG group initially had a more severe functional deficit, which was further supported by the evaluated temporal parameters of the GC, kinematics, and EMG. The differences were slightly smoothed by the training and treatment, although they did not disappear completely. This case has clearly demonstrated a non-equivalence of clinical and functional assessments: while having similar clinical characteristics, the two patient groups differed significantly in their gait function, with the SSPG group clearly walking worse and slower, and with greater functional asymmetries.

The post-treatment stride length in the SSPG group was smaller than that in the SPG group before treatment, which also confirms more severe functional deficits in the SSPG group.

A most interesting point is the differences in the actually trained parameter. In both patient groups, significant post-treatment changes in the SP were noted only on the unaffected side. In the SPG group, the SSP on the affected side significantly increased as a result of training. These clearly favorable changes were found only in the SPG group. In the SSPG group, slightly worse results were obtained for all parameters except for the DSP, even before training. The SP showed a significant difference for both lower extremities, while SSP, only for the contralateral one. In the SSPG group, the DSP was also longer, which was indicative of a more severe impairment. Thus, clinically, there were no differences between the patient groups before the study treatment, yet patients in the SSPG group still showed a significantly worse gait function than those in the SPG group.

A direct comparison of our findings with any similar studies was not possible because no such studies are available so far. The previously cited study by Kantan et al. [23] explored the possibility of using an audio BFB with swing phase as the target parameter. This is close to our study because the stance phase and the swing phase are directly related with each other within the gait cycle. Furthermore, the single support phase of one leg coincides in time with the swing phase for the other. However, that study differed from ours in design and objective. The authors reported positive changes in three of nine patients. In another study, Giraldo-Pedroza et al. [41] used BFB training in seven elderly subjects to increase the swing phase duration. The results were positive, with the phase duration increased by 6.5%. In that study, however, the authors also had a different objective and the subjects had no functional gait disorders but only age-related gait changes.

It should also be noted that in both groups the best response to training was on the contralateral side. The reason is obvious: the unaffected limb allows full control and, consequently, more effective training.

The kinematic data confirmed that the SSPG group had a significantly lower gait function compared to the SPG group. Positive changes were noted in the knee joint movements in the SPG group, with a significant increase in the Ka1. The Ka2 increased at the end of treatment in both groups. This was a positive sign of prevention of a further development of passive closure of the knee joint. The main flexion amplitude Ka3 also increased significantly but only in the SSPG group. Thus, positive changes in different amplitudes were noted in both patient groups. However, the swing amplitude (Ka3) was the most important for the movement.

The range of motion in the ankle joint showed no changes in either group; however, the groups did not include any patient with a manifest pathology such as foot drop.

The EMG muscle activity also showed no change at the end of treatment. It was expectable for this phase of the disease and the relatively short treatment period.

The novelty of the presented study consists in BFB training based on a single target biomechanical parameter. Of the two target parameters used in the study, the SP was associated with more significant changes, especially on the unaffected side. Perhaps, a more severe functional deficit of patients in the SSPG group—which was not evident from the clinical assessments used to select patients for the study—could have played a role.

This study has shown that, in the future, groups of patients should be formed based on biomechanical gait parameters rather than clinical ones. Only this selection method allows the formation of functionally equivalent groups. It is probably necessary to review the severity criteria of a patient’s functional gait impairment for BFB training. A premature start to training may reduce its effectiveness. It is also possible that unaffected leg parameters make better BFB targets. They are the ones that can be trained due to preserved central control.

## 5. Conclusions

The clinical assessments significantly differed from those based on an instrumental gait analysis. It would be more correct to form patient groups based on gait analysis data.

Targeted training of gait function can be administered in subacute stroke patients.

BFB training with the SP as the target parameter is probably preferable. According to the study data, the unaffected leg responds to the training. As the SP on the unaffected side decreases, the load on the paretic side grows, and the gait symmetry improves. Thus, the training objective is still being achieved.

To achieve a better effect, the amount of BFB training should be considerably larger than the amount in the study we have conducted.

## Figures and Tables

**Table 1:** Characteristics of the study groups.

Group	Number (Males/Females	Hemiparesis Right/Left	Age (Years)	Height (sm)	Weight (kg)	Days After Stroke
SPG	15/5	9/11	49.0 ± 12.4 (23–65)	176.3 ± 8.5 (159–195)	79.5 ± 13.3 (49–102)	113.0 ± 47.9 (28–179)
SSPG	10/10	11/9	55.0 ± 11.5 (35–74)	174.4 ± 8.1 (159–187)	78.9 ± 10.8 (59–95)	100.7 ± 58.6 (25–179)
Control	10/10	-	28.8 ± 3.7 (23–35)	176.8 ± 5.5 (168–188)	76.2 ± 14.1 (55–100)	-

**Table 2:** Functional assessment outcome scales and capability scales.

Scale	SPG		SSPG	
	Before	After	Before	After
Barthel Index	74.7 ± 10.3	81.7 ± 10.3 *	73.7 ± 8.4	85.5 ± 10.5 *
Rivermead	9.4 ± 2.1	11.0 ± 2.1 *	8.8 ± 1.9	10.8 ± 1.7 *
Rankin Scale	3.0 ± 0.0	2.8 ± 0.4 *	3.0 ± 0.0	2.6 ± 0.7 *
RRS	3.0 ± 0.3	2.8 ± 0.4 *	3.0 ± 0.0	2.6 ± 0.7 *
Berg Balance	46.9 ± 4.6	50.2 ± 3.9 *	39.8 ± 5.9 ^†^	45.8 ± 5.9 *^†^
FIM	12.6 ± 0.8	12.8 ± 0.9 *	12.0 ± 1.5	12.3 ± 1.4 *
TUG	22.5 ± 6.3	18.0 ± 5.4 *	25.3 ± 7.1	20.0 ± 7.5 *
Hauser Index	3.3 ± 0.4	2.7 ± 0.7 *	3.1 ± 0.8	2.9 ± 0.6 *

*—significant, *p* < 0.05 compared to the same parameter before the treatment; ^†^—significant, *p* < 0.05 compared to the same parameter in the SPG group.

**Table 3:** Spatial and temporal gait parameters.

Parameter	Group	Before		After		Control
		Contralateral	Paretic	Contralateral	Paretic	
GC, s	SPG	1.6 ± 0.3 *	1.6 ± 0.3 *	1.5 ± 0.33 *	1.5 ± 0.3 *	1.1 ± 0.1
	SSPG	1.8 ± 0.5 *	1.7 ± 0.5 *	1.8 ± 0.42 *	1.7 ± 0.4 *	
Cadence, steps/min	SPG	39.8 ± 7.3 *		40.4 ± 7.4 *		54.6 ± 3.5
	SSPG	35.4 ± 9.9 *		35.5 ± 9.2 *		
Cl, cm	SPG	12.2 ± 1.8	9.9 ± 3.1 *^#^	12.6 ± 1.6	10.2 ± 3.0 *^#^	12.5 ± 2.1
	SSPG	10.1 ± 2.8 *^&^	8.3 ± 3.3 *^#^	10.6 ± 1.9 *^&^	9.3 ± 2.5 *^$^	
V, km/h	SPG	2.1 ± 0.8 *		2.3 ± 0.9 *^$^		4.4 ± 0.6
	SSPG	1.4 ± 0.8 *^&^		1.6 ± 0.8 *^&^		
SL, cm	SPG	85.4 ± 21.7 *		90.6 ± 25.4 *^$^		135.6 ± 11.2
	SSPG	63.2 ± 24.4 *^&^		71.5 ± 22.4 *^&$^		
SP, %	SPG	72.8 ± 5.6 *	63.5 ± 4.3 ^#^	71.2 ± 4.9 *^$^	64.5 ± 3.6 ^#^	62.8 ± 1.5
	SSPG	76.4 ± 9.8 *^&^	69.5 ± 7.4 *^&#^	74.1 ± 9.8 *^$^	68.4 ± 8.2 *^&^	
SSP, %	SPG	36.1 ± 4.3	27.1 ± 6.0 *^#^	35.8 ± 3.6	29.1 ± 4.7 *^#$^	37.2 ± 1.4
	SSG	31.0 ± 6.6 ^&^	24.2 ± 9.2 ^#^	32.3 ± 8.2	26.5 ± 9.3 ^#^	
DSP, %	SPG	36.7 ± 8.3 *	36.4 ± 8.0 *	35.4 ± 6.8 *	35.3 ± 6.7 *	25.6 ± 2.7
	SSPG	44.2 ± 14.7 *^&^	43.6 ± 15.0 *^&^	41.9 ± 15.9 *	42.1 ± 16.1 *	

*—significant, *p* < 0.05 (compared to the same parameter in the control group); ^#^—significant, *p* < 0.05 (compared to the same parameter on the contralateral side); ^$^—significant, *p* < 0.05 (compared to the same parameter before the treatment); ^&^—significant, *p* < 0.05 (compared to the same parameter in the SPG group).

**Table 4:** Kinematic parameters of movements in the hip, knee, and ankle joints (in degrees).

Parameter	Group	Before		After		Control
		Contralateral	Paretic	Contralateral	Paretic	
HA	SPG	29.7 ± 3.8 *	24.3 ± 5.6 *^#^	31.1 ± 4.9	24.1 ± 5.9 *^#^	33.2 ± 4.8
	SSPG	25.5 ± 6.7 *^&^	17.8 ± 6.4 *^#&^	26.1 ± 5.2 *^&^	18.8 ± 6.9 *^#&^	
Ka1	SPG	7.3 ± 4.3 *	5.8 ± 4.6 *	7.1 ± 5.7 *	8.1 ± 5.6 *^&^	14.6 ± 3.5
	SSPG	5.7 ± 4.8 *	5.6 ± 4.2 *	5.0 ± 4.3 *	6.5 ± 5.1 *	
Ka2	SPG	−2.1 ± 9.1 *	−6.1 ± 6.6 *^#^	−2.7 ± 10.7 *	−1.7 ± 7.5 *^$^	4.8 ± 4.8
	SSPG	−0.5 ± 6.9 *	0.4 ± 6.6 *^&^	−2.3 ± 9.0 *	1.8 ± 6.1 ^#&^	
Ka3	SPG	45.2 ± 9.9 *	25.4 ± 12.9 *^#^	47.3 ± 10.3 *	29.0 ± 14.6 *	56.50 ± 7.33
	SSPG	39.8 ± 11.4 *	27.0 ± 13.6 *^#^	40.4 ± 10.8 *^&^	32.3 ± 16.4 *^#$^	
AA	SPG	25.8 ± 5.6 *	22.4 ± 8.3 *^#^	26.7 ± 6.0 *	22.8 ± 6.2 *^#^	33.4 ± 6.2
	SSPG	21.8 ± 4.5 *^&^	19.9 ± 6.9 *	23.0 ± 7.0 *	23.7 ± 13.2 *	

*—significant, *p* < 0.05 (compared to the same parameter in the control group); ^#^—significant, *p* < 0.05 (compared to the same parameter on the contralateral side); ^$^—significant, *p* < 0.05 (compared to the same parameter before the treatment); ^&^—significant, *p* < 0.05 (compared to the same parameter in the SPG group).

**Table 5:** EMG data.

Muscle	Group	Before		After		Control
		Contralateral	Paretic	Contralateral	Paretic	
TA	SPG	141.3 ± 56.4	110.4 ± 59.5 *^#^	132.7 ± 48.3	111.2 ± 54.5 *	139.6 ± 38.4
	SSPG	148.9 ± 66.5	108.6 ± 67.0 *^#^	144.8 ± 65.8	111.0 ± 59.6 *^#^	
GA	SPG	115.7 ± 60.6	54.4 ± 35.3 *^#^	106.8 ± 54.1	69.0 ± 52.8 *^#^	118.0 ± 43.8
	SSPG	106.6 ± 43.3	64.2 ± 51.0 *^#^	112.4 ± 56.2	62.7 ± 37.0 *^#^	
QA	SPG	57.3 ± 23.2	50.5 ± 39.2	73.5 ± 30.6	57.0 ± 37.5	64.8 ± 45.5
	SSPG	55.6 ± 31.6	50.9 ± 29.2	46.2 ± 20.9	50.4 ± 28.4	
HA	SPG	82.2 ± 39.8	52.0 ± 36.1 *^#^	82.5 ± 40.2	48.3 ± 33.2 *^#^	75.5 ± 33.1
	SSPG	70.3 ± 35.3	45.2 ± 28.5 *^#^	61.5 ± 33.2	41.6 ± 27.4 *^#^	

*—significant, *p* < 0.05 (compared to the same parameter in the control group); ^#^—significant, *p* < 0.05 (compared to the same parameter on the contralateral side).

## Data Availability

The data used in this study are available at DOI: 10.17632/8f4mpm9w2z.1.
